# The return to normal life: sleep, anxiety upon awakening, and nightmares following the crisis caused by the COVID-19 pandemic

**DOI:** 10.3389/frsle.2026.1748727

**Published:** 2026-06-24

**Authors:** Inigo Saez-Uribarri

**Affiliations:** Independent Researcher, Sopela, Spain

**Keywords:** COVID-19, dream content, emotional processing, mental health, morning anxiety, nightmares, post-pandemic recovery, sleep quality

## Abstract

**Introduction:**

The return to normal life following the COVID-19 pandemic was evaluated in terms of anxiety upon awakening, sleep quality indicators, and dream characteristics.

**Methods:**

The sample comprised 394 women and 107 men who completed an online self-report questionnaire between August 15, 2022, and December 8, 2022 about their sleep and dream experience from the previous night. Respondents completed the Anxiety upon Awakening Assessment Questionnaire (CEAD, as per the Spanish acronym) and evaluated their experiences of the pandemic. The data were compared to those obtained before the pandemic and those collected during lockdown in a previous study.

**Results:**

After the return to normal life, 46.5% of respondents recalled at least one dream scene, and nightmares were recorded in 4.6% of cases. This percentage did not differ significantly from those observed during lockdown or before the pandemic. Compared with the previous periods, participants also reported more dreams with anxious content and greater dream recall. A return to a sleep duration of between 6 and 7 h was observed. However, anxiety upon awakening was higher, suggesting that full normalization had not yet occurred at the time of this study.

**Conclusion:**

In this sample, nightmare frequency was less sensitive than anxiety upon awakening to post-pandemic sleep-related emotional activation. The findings may be compatible with emotional-processing accounts of dreaming and with a homeostatic interpretation of sleep in which emotional dream content did not translate into more awakenings.

## 1 Introduction

The COVID-19 pandemic provided an unexpected context for examining psychological adaptation to a global threat to survival and the subsequent return to normal life. This threat affected populations worldwide (Mota et al., 2020; Sikka et al., 2024; Yang et al., 2023), triggered psychological distress (Aknin et al., 2022; Kilius et al., 2021; Solomonova et al., 2021), and disrupted daily life, especially social interaction and mental health (Schredl and Bulkeley, 2020). Although public health crises have historically been associated with increased anxiety, sleep disorders and dream characteristics have received comparatively less attention as indicators of psychological adaptation and recovery. A more detailed examination is therefore needed of how sleep, dreams, and anxiety-related awakening experiences changed after the acute phase of the crisis. The present study pursues two aims: first, to evaluate the return to normal life in terms of anxiety upon awakening (AUA), sleep quality, and dream characteristics; and second, to compare these data with those of a previous baseline study (Saez-Uribarri, 2020b), which included data from before the pandemic and during lockdown. In both studies, nightmares were assessed using a specific operational definition intended to distinguish them more clearly from other negative or disturbing dream experiences.

In the baseline study, no increase in nightmare frequency was identified during strict lockdown. This finding was interpreted in relation to lower dream recall, the absence of greater self-reported fear or anxiety caused by dreams, and fewer recurring dream elements. However, AUA increased during lockdown and was associated with COVID-19-related worry, belonging to a risk group, and perceiving the dream as a nightmare. To extend these findings, the same type of data was collected again in a new sample after pandemic restrictions had been lifted in Spain.

Previous studies provide a strong rationale for examining post-lockdown sleep and dreaming, as they reported poorer sleep quality, increased anxiety, increased perceived nightmares, vivid dream recall, and more negatively toned dream content during lockdown (Kilius et al., 2021; Pesonen et al., 2020; Scarpelli et al., 2022; Sikka et al., 2024; Simões et al., 2023; Solomonova et al., 2021; Westerberg et al., 2024). Qualitative studies also highlighted the emotional relevance of pandemic-related dreaming, including threat-related material, mourning-related themes, explicit COVID-19-related content, anguish, isolation, aggression, and vulnerability (Margherita et al., 2021; Monaco et al., 2022; Sacchetti et al., 2024). However, other authors did not find substantial changes in dream recall, emotionality, vividness, or dream themes (Koppehele-Gossel et al., 2023; Scarpelli et al., 2022; Schredl and Bulkeley, 2020; Sikka et al., 2024; Yang et al., 2023), indicating that the effect of the pandemic on dreaming was neither simple nor uniform across studies or populations.

Beyond these general findings, pandemic-related threatening dreams and nightmares were associated with several individual and contextual factors, such as being female or younger, traumatic experiences, elevated stress, loneliness related to lockdown, and sleep changes, especially poorer sleep quality (Koppehele-Gossel et al., 2023; Pesonen et al., 2020; Sikka et al., 2024; Simões et al., 2023; Solomonova et al., 2021; Westerberg et al., 2024). Algorithm-based content analysis indicated a direct effect of the pandemic on dream content independent of perceived stress (Pesonen et al., 2020). Intensive media exposure was also associated with a higher frequency of threatening dreams, mediated by anxiety and low coping efficacy (Guo and Shen, 2021). Sikka et al. (2024), in line with the continuity hypothesis of dreaming, found that worse sleep quality was associated with more negative affect in dreams and a greater likelihood of reporting a nightmare. They also found that average worry, but not day-to-day worry, was associated with more negative dream affect, indicating that more worry-prone profiles may have been especially vulnerable.

Anxiety was a central psychological response during lockdown and was frequently examined in relation to sleep disturbance, dream activity, and nightmare frequency. During lockdown, anxiety reflected the social climate generated by the crisis and by constant media exposure (Guo and Shen, 2021). Elevated anxiety levels were reported during this period (Casagrande et al., 2020; Huang and Zhao, 2020; Koppehele-Gossel et al., 2023; Saez-Uribarri, 2020b), together with more dreams and nightmares among individuals with anxiety (Solomonova et al., 2021). In Solomonova et al. (2021), anxiety was predicted by dream frequency, bad dreams, nightmares, and frequent pandemic-related dream contents, especially inefficacy, physical threat, the pandemic itself, and death.

A particularly relevant moment for anxiety in relation to dreaming is awakening, when the emotional content of dreams may remain salient. AUA is understood here as a predominantly anxiety-related emotional state that emerges during the transition from sleep to wakefulness, rather than as a measure of general or trait anxiety. Previous studies (Saez-Uribarri, 2008; Saez-Uribarri and Oberst, 2020) have examined this construct specifically in relation to awakening after dream activity, indicating that it may capture a distinct aspect of the awakening experience. In this sense, AUA is intended to reflect the immediate emotional tone associated with sleep and nocturnal dream experience, distinguishing it from broader constructs such as general morning mood, negative affect, or non-emotional post-sleep states such as sleep inertia. Its potential added value lies in providing information about the emotional impact of sleep and dreams that is not fully captured by nightmare frequency alone. This study therefore analyzes sleep duration, AUA, and nightmare frequency together to determine whether these parameters returned to normal after the lifting of restrictions or whether post-pandemic emotional activation remained detectable.

The lockdown caused by the COVID-19 pandemic also served as a natural context for comparing theoretical approaches to the function of dreams, particularly the wakefulness-dreaming continuity hypothesis, threat simulation theory, and emotional-processing frameworks (Mota et al., 2020; Proença Becker et al., 2024). According to the continuity hypothesis, daytime stimuli and stress are projected into dream content (Domhoff, 1996; Schredl, 2003), which may help explain the increase in threatening or disturbing dream experiences during the crisis (Schredl and Bulkeley, 2020) and the finding that dreams during the first phase of the pandemic reflected daytime pandemic experiences (Sacchetti et al., 2024). The pandemic also provided an opportunity to examine simulation theories that emphasize environmentally threatening signals, which are ethically impossible to reproduce under experimental conditions (Abbas and Samson, 2023). Emotional-processing accounts, in turn, propose that dream activity may contribute to the further elaboration or regulation of waking emotions (Nielsen et al., 1991; Nielsen and Lara-Carrasco, 2007), an idea that has been invoked to explain pandemic-related changes in dreaming (Mota et al., 2020; Proença Becker et al., 2024; Solomonova et al., 2021).

Even though a large number of studies already exist on the effect of the pandemic on dream content, emotionality, and nightmares, additional information is needed beyond that provided by retrospective studies, whose reliability is limited. According to Sikka et al. (2024), such studies may not provide reliable data on affect and dream frequency, and they argue that data should be collected at the time of occurrence. Loukola et al. (2024) likewise argued that retrospective studies may collect the impression people have of these measures, rather than more accurate information about content and frequency. While such designs make it possible to obtain large samples, they also introduce memory biases (Alfonsi et al., 2022; Scarpelli et al., 2022). In addition, data-collection methods and operational definitions of nightmares varied widely across studies. In some, participants were not given specific instructions to determine whether they had experienced a nightmare (Kilius et al., 2021; Proença Becker et al., 2024; Simões et al., 2023; Solomonova et al., 2021). Other studies used more specific procedures, such as questionnaires based on the DTS content-analysis scale, definitions of nightmares as recurrent disturbing dreams, classifications based on self-reported dream content, or the use of the MADRE questionnaire (Schredl et al., 2014) with an explicit definition of nightmare (Guo and Shen, 2021; Jiang et al., 2020; Musse et al., 2020; Revonsuo and Valli, 2008; Westerberg et al., 2024). These methodological differences may substantially influence both the apparent frequency of nightmares and the interpretation of dream emotionality.

Against this background, Westerberg et al. (2024) noted that it remains unclear whether changes in sleep and dreams persisted beyond the lockdown period or after people had psychologically adapted to COVID-19. This is especially noteworthy given that many subjective components of psychological wellbeing had recovered by mid-2020 (Aknin et al., 2022), whereas prospective or same-day studies on pandemic-related dreams remain scarce (Loukola et al., 2024; Sikka et al., 2024). To address this gap, the present study compares post-lockdown sleep and dream data with pre-pandemic and lockdown data from an earlier study. Its geographical scope was restricted to Spain, where the population experienced a particularly intense social, political, and media environment. The national response included a strict lockdown followed by a gradual lifting of restrictions, and by August 2022 normal life appeared to have resumed (Ministerio de Sanidad, 2022). At that point, it was reasonable to expect sleep and dream characteristics to move back toward pre-pandemic parameters.

This study therefore examines whether those parameters returned to pre-pandemic levels or remained altered after the COVID-19 crisis. To test this, data were collected from a sample of participants between August and December 2022 and compared with those of the baseline study (Saez-Uribarri, 2020b). The hypotheses are based on the premise that the stressful events and uncertainty generated by the COVID-19 crisis may have influenced sleep and dream patterns, while also allowing recovery once the acute phase had passed. This allowed for the evaluation of the following hypotheses:

- H1: Sleep duration following lockdown would be reduced compared to the increase observed during lockdown and would not be significantly different from the period prior to lockdown.
- H2: AUA would show lower values with respect to the increase observed during lockdown and would not be significantly different from the period prior to lockdown.
- H3: Based on the results of the baseline study, nightmare frequency would not be expected to change significantly with the return to normal life compared to the lockdown period.

## 2 Materials and methods

A repeated cross-sectional design was used, given that the use of anonymous data made it impossible to follow the same sample longitudinally. Participants were given an online questionnaire once the restrictions on movement and social gatherings due to the virus had been lifted throughout Spain. The data collection period was between August 15, 2022, and December 8, 2022. The questionnaire is available in the Supplementary material.

The study protocol was approved by the Clinical Research Ethics Committee of the OSI Ezkerraldea-Enkarterri-Cruces (code E20/37). Participation was voluntary and anonymous. Before completing the online questionnaire, participants were informed about the aims of the study, the anonymous processing of the data, and that submission of the questionnaire implied informed consent to participate. The study was conducted in accordance with Spain's Law 14/2007 on Biomedical Research, Spain's Organic Law 3/2018 on the Protection of Personal Data and Guarantee of Digital Rights, and Regulation (EU) 2016/679 of the European Parliament and of the Council.

### 2.1 Participants

The inclusion criteria were that participants' first language was Spanish, that they resided in Spain, that they were over 18 years of age, and that they had not previously completed the questionnaire used in earlier studies. Compliance with the language requirement was assumed when participants had been born in a Spanish-speaking country. Participants were recruited through a Facebook advertisement targeted at people living in Spain. Because the data were anonymous, individual participants from earlier waves could not be tracked directly; accordingly, the post-lockdown sample was treated as an independently recruited repeated cross-sectional cohort rather than as a longitudinal follow-up. To minimize overlap with previous datasets, respondents were asked whether they had already completed the questionnaire in a previous study, and those who answered affirmatively were excluded. Probable duplicate cases were also excluded when the same date of birth, sex, and birthplace were recorded. Each individual contributed only one questionnaire to the final dataset. In total, 40 cases were excluded. The final sample comprised 501 participants: 107 men (mean age = 53.8, SD = 14.8) and 394 women (mean age = 47.0, SD = 14.0), with ages ranging from 18 to 84 years. Women were overrepresented in the sample, and the age difference between men and women was significant according to the Mann-Whitney *U* test, *U* = 15479.00, *z* = −4.22, *p* < 0.001.

### 2.2 Measures

#### 2.2.1 Dreamer profile, medication, and sleep disorders

Information was recorded on participants' sex, age, and their date, place, and country of birth. They were also asked if they had completed the questionnaire previously, for their total sleep time, and how long had passed between the participant waking up and completing the questionnaire. To complete the profile data, they were asked about taking sleep or other medication, and about sleep disorders diagnosed by a doctor. These data were collected to control for possible biases relating to sleep, nightmares, and AUA.

#### 2.2.2 The anxiety upon awakening assessment questionnaire (CEAD)

An abridged 19-item version of the Anxiety upon Awakening Assessment Questionnaire (CEAD; Spanish acronym for *Cuestionario de Evaluación de la Ansiedad al Despertar*) was used to assess participants' anxiety at the time of awakening. The online questionnaire included the original set of awakening items, but the CEAD score analyzed in this study was computed using the validated 19-item abridged scoring scheme. The original instrument comprised 25 items, but prior psychometric work showed that the initial 25-item model did not provide satisfactory fit. A reduced 19-item version showed adequate fit in confirmatory factor analysis (CMIN/DF = 2.91, GFI = 0.91, CFI = 0.94, RMSEA = 0.06) and good internal consistency (Cronbach's α = 0.91) (Saez-Uribarri, 2020a). The retained items loaded on a single factor of anxiety upon awakening, supporting the construct validity of the abridged version. Accordingly, this version was used in the present study. The CEAD has previously been used as a criterion variable in studies examining dream content (Saez-Uribarri, 2008; Saez-Uribarri and Oberst, 2020). Each item is rated on a 5-point response scale reflecting increasing intensity of the sensation upon awakening. As described in the confirmatory factor analysis, the response categories were transformed using the non-linear Rankit method and multiplied by the standardized beta coefficient of each item. The total CEAD score is obtained by summing, across the 19 items, the score corresponding to the selected response category, with higher total scores indicating greater anxiety upon awakening. For example, the item “Upset” is scored using the following values: −0.35, 0.52, 0.84, 1.19, and 1.73. The full scoring scheme is provided in the Supplementary Material Table S5. In the present study, Cronbach's alpha for the CEAD was 0.94.

#### 2.2.3 Evaluation of sleep and dreams

All participants were questioned about their sleep duration, the stimulus that woke them, and their recall of their dream. Sleep duration was evaluated with an ordinal variable. The options for the awakening stimulus were: “Alarm clock”, “The person who shares a bedroom with me”, “Noise in my surroundings”, “What I was dreaming about”, and “Something else”. For dream recall, the values were: “I could not remember anything”, “Only the impression of having been dreaming”, “An image, sound, sensation, smell (without there being a story)”, “A simple scene/something was happening”, “A dream with several scenes/several things were happening”, and “I remember several different dreams during the night”.

People who recalled their dreams were asked about recurring elements, the feeling of fear or anxiety caused by the dream, and if they considered it to be a nightmare, without providing a definition of this. Regarding the feeling of fear or anxiety caused, the options were: “Fear”, “Anxiety”, “Fear and Anxiety”, “Another feeling”, and “I don't know”.

#### 2.2.4 Evaluation of nightmares

In this study, a nightmare was defined as a dream in which the participant gave the following responses to three separate items on the questionnaire:

- They remembered at least a simple scene from the dream. This condition is based on the need for the participant to remember a plot to differentiate them from other dream events, such as night terrors.
- The dream caused fear, anxiety, or both. This criterion acknowledges the need for an emotional reaction typical of nightmares to be produced in the participant.
- What they were dreaming about woke them up. This component emphasizes that the dream was disturbing enough to interrupt their normal cycle, with awakening considered to be a possible adaptive response of the body to the disturbing experience of the dream.

If participants gave affirmative responses to all three questions, Nightmare was given a value of 1; otherwise, its value was 0. This definition sought to prevent participants from being the ones to decide whether to classify their dream as a nightmare or not. In other studies, no definition of a nightmare has been given to participants, or one has been given, and they were the ones to make the final decision on the matter. In this study, it was decided to break down the question into three elements to increase the reliability of the definition: a memory element, an emotional element, and a disruptive element. It was believed that a self-assessment of these three simpler elements would improve the evaluation of nightmares.

To make comparisons, participants were also asked if, in their view, they had experienced a nightmare, without giving them prior information or a definition of the concept; that is, based on their own interpretation of the term.

#### 2.2.5 Perception and behavior in response to COVID-19

Several questions were included to better understand the context of worry, risk perception, and changes in habits. Participants were asked how many times they had felt worried about the crisis in the last day, how intense that worry was, if they considered themselves to be part of a risk group, and what precautions they were taking to avoid infection.

### 2.3 Data analysis

The confidence level used for all statistical analyses was 95%. To compare the post-lockdown data with those of the baseline study (Saez-Uribarri, 2020b), a dummy variable called Group was created with three values: Pre, COVID-19, and Post. To make the COVID-19 group more homogeneous, participants not resident in Spain were excluded from the baseline dataset. The Pre (*n* = 483, women = 72.9%, mean age = 39.4, SD = 12.9) and COVID-19 (*n* = 456, women = 71.5%, mean age = 49.5, SD = 12.2) data came from the baseline study, whereas the Post data were collected in the present study.

The ordinal variables total sleep time and time elapsed between awakening and questionnaire completion were subjected to Tukey's rank-based inverse normal transformation to obtain values approximating a normal distribution. Distributional assumptions were evaluated using the Kolmogorov-Smirnov test (D). Because CEAD scores were not normally distributed, they were log10-transformed to improve symmetry. When the assumptions for the planned analyses were not met, bootstrapping with 1,000 replicates was used. In selected contingency analyses with sparse expected frequencies, Monte Carlo estimates were used for the chi-square significance test.

In the bivariate analyses, categorical variables were examined using chi-square tests and standardized residuals, and effect size was summarized with Cramér's *V*. Associations involving ordinal or non-normally distributed variables were examined using Spearman's rho (ρ). Non-parametric group differences were examined using the Kruskal-Wallis test, and effect size was summarized with epsilon-squared (ε^2^). For nightmare frequency, pairwise absolute differences in proportions between groups were also calculated, and 95% confidence intervals were estimated using stratified bootstrapping with 10,000 resamples to assess the magnitude and precision of between-group differences.

To test hypothesis H1 regarding total sleep time, an analysis of covariance (ANCOVA) was conducted with Group and Sex as factors and Age as a covariate (Supplementary Table S2). Homogeneity of variance was assessed with Levene's test, homogeneity of regression slopes was examined by testing the Sex × Age and Group × Age interactions, pairwise comparisons of adjusted marginal means were Bonferroni-adjusted, and effect size was reported as partial eta squared ($\eta_{\text{p}}^{2}$). To test hypothesis H2 regarding AUA, CEAD scores were compared across the three groups using a bootstrap-based analysis of variance complemented with Scheffé *post hoc* tests, and linear regression models were then estimated; model fit was summarized with *R*^2^, and residual independence was examined with the Durbin-Watson statistic. To test hypothesis H3 regarding nightmares, logistic regression models were estimated; odds ratios with 95% confidence intervals were examined, and model performance was summarized with Nagelkerke's *R*^2^, the area under the curve (AUC), and the Hosmer-Lemeshow test.

Finally, a path analysis was conducted to obtain an integrated model of the findings. Model fit was evaluated using χ^2^, CMIN/DF, CFI, TLI, NFI, and RMSEA. Because multivariate normality was not satisfied, as indicated by Mardia's test, the model was also recalculated using the distribution-free least squares discrepancy procedure.

## 3 Results

### 3.1 Data on the return to normal life

Firstly, the basic sleep and health variables indicated that the median sleep duration was between 6 and 7 h. Sleep medication was taken by 13.0% of participants, and 33.3% used other types of medication. Regarding the awakening stimulus, 26.3% of participants were woken by their alarm clock, 16.6% by noise, and 6.8% by the person sharing a bedroom with them.

Secondly, dream recall and the three components used to define nightmares were examined separately. In the Post sample, 310 participants recalled some dream content and answered the subsequent dream-related questions. Of the full Post sample, 233 participants (46.5%) recalled at least one dream scene, meeting the recall criterion required for the operational definition of nightmare. Among participants who recalled any dream content, 121 of 310 (39.0%) reported fear, anxiety, or both in response to the dream. In the full Post sample, 41 participants (8.2%) reported that they had awakened because of what they were dreaming. Finally, 23 participants (4.6%) met all three criteria simultaneously—recall of at least one dream scene, fear or anxiety caused by the dream, and awakening caused by dream content—and were therefore classified as having had a nightmare according to the operational definition used in this study. This percentage contrasted with the percentage of perceived nightmares, which was higher: 54 participants, corresponding to 10.8% of the full Post sample, considered their dream to have been a nightmare. In addition, 11 participants (2.2%) reported an almost identical or recurrent dream, whereas 72 participants (14.4%) recognized some repeated dream elements.

### 3.2 Nightmares with the return to normal life

In general, the control variables examined in the study were not clearly associated with nightmares. The only exception was sleep medication, which was associated with a lower likelihood of reporting a nightmare (ρ = −0.17, *p* < 0.001). Pandemic-related variables, such as belonging to a risk group or engaging in behaviors to avoid COVID-19, were not associated with nightmares. Nor were sleep duration, sleep disorders, or the frequency of worry about COVID-19. However, nightmare frequency showed a small positive association with the intensity of worry, ρ = 0.10, *p* = 0.031.

Furthermore, a significant association was observed between nightmares and perceived nightmares, χ^2^(2) = 33.98, *p* < 0.001, Cramér's V = 0.33, whereas no association was found between nightmares and recurring content, χ^2^(4) = 3.69, *p* = 0.38, Cramér's V = 0.11; in both cases, chi-square significance was estimated using the Monte Carlo procedure. Meanwhile, nightmare frequency was associated with the intensity of worry, χ^2^(3) = 8.40, *p* = 0.034, Cramér's V = 0.13. Finally, the relationship between nightmares and the CEAD score was notable, ρ = 0.18, *p* < 0.001.

### 3.3 Anxiety upon awakening with the return to normal life

AUA, as measured with the CEAD, turned out to be more sensitive than nightmares for generating differences or relationships with other variables. With respect to the control variables, a positive relationship was found between the CEAD scores in women (ρ = 0.09, *p* = 0.033) and a negative one with age (ρ = −0.17, *p* < 0.001), sleep time (ρ = −0.22, *p* < 0.001), and sleep medication, ρ = −0.28, *p* < 0.001. No memory biases were apparent, with a non-significant relationship between scores on the CEAD and time before completing the questionnaire, ρ = −0.05, *p* = 0.226.

With regard to sleep quality, there was a negative relationship between the CEAD scores and not having been diagnosed with a sleep disorder, ρ = −0.24, *p* < 0.001. People who sleep better appear to have lower CEAD scores. In keeping with this pattern, the 59 people with insomnia (ρ = 0.23, *p* < 0.001) and the 18 with restless legs syndrome (ρ = 0.13, *p* = 0.003) had higher CEAD scores.

In terms of dreams, better dream recall was not related to the CEAD scores, but it was related to recurring content, *H*(4) = 20.67, *p* < 0.001, ε^2^ = 0.05. Also, perceived nightmares generated differences with the CEAD scores (*H*(2) = 29.05, *p*<*0.0*01, ε^2^ = 0.09), with higher values for those who had classed their dream as a nightmare and among those who said they did not know if their dream was a nightmare.

Regarding COVID-19-related variables, belonging to a risk group was not generally associated with CEAD scores. The exception was participants who considered themselves at risk because of age, who showed lower CEAD scores (ρ = −0.09, *p* = 0.041). This association may reflect the concurrent relationship with age, which was negatively associated with CEAD scores. Behaviors to avoid infection were not uniformly associated with CEAD scores. Frequent hand-washing and the use of masks, gloves, or hand sanitizer were not associated with significant differences. In contrast, higher CEAD scores were observed among participants who tried not to touch potentially contaminated surfaces (ρ = 0.12, *p* = 0.009) and those who tried not to leave home without a good reason (ρ = 0.11, *p* = 0.009). CEAD scores also showed positive associations with the frequency of worry about COVID-19 (ρ = 0.28, *p* < 0.001) and its intensity (ρ = 0.24, *p* < 0.001).

### 3.4 Comparison with the baseline study

An ANCOVA was conducted with total sleep duration as the dependent variable, Group (Pre, COVID-19, Post) and Sex as factors, and Age as the covariate. Homogeneity of variance was confirmed (Levene's *p* = 0.103), and no evidence of violation of the homogeneity of regression slopes assumption was found (Sex × Age: *p* = 0.971; Group × Age: *p* = 0.099). In the final model reported in Supplementary Table S2, there was a significant effect of Group on sleep duration, *F*(2, 1419) = 31.34, *p* < 0.001, η^2^*p* = 0.04, whereas no significant effect of Sex was observed, *F*(1, 1419) = 2.01, *p* = 0.156. Bonferroni-adjusted pairwise comparisons showed that the Pre and Post groups did not differ significantly (Δ = 0.10, *p* = 0.366), whereas the COVID-19 group showed higher adjusted sleep duration than the Pre (Δ = 0.50, *p* < 0.001) and Post (Δ =0.40, *p* < 0.001) groups. These results support hypothesis H1 that sleep duration returned to pre-pandemic levels once the public health restrictions were lifted.

Regarding nightmares, the comparison across the Pre, COVID-19, and Post groups showed no significant differences in nightmare frequency, χ^2^(2) = 2.00, *p* = 0.367, Cramér's V = 0.04. The observed percentages were 3.7% in the Pre group, 2.9% in the COVID-19 group, and 4.6% in the Post group. Taking the three groups together, the overall percentage of nightmares was 3.8%, 95% *CI* [2.8, 4.7], calculated by bootstrapping stratified by Group. Pairwise bootstrap estimates of absolute differences in proportions also indicated small differences between periods: −0.88 percentage points for COVID-19 vs. Pre, 95% *CI* [−3.20, 1.40]; 1.74 percentage points for Post vs. COVID-19, 95% *CI* [−0.59, 4.07]; and 0.86 percentage points for Post vs. Pre, 95% *CI* [−1.58, 3.32]. These intervals included zero in all comparisons, indicating that the pairwise estimates were not statistically distinguishable from zero. These results support hypothesis H3, which stated that nightmare frequency would not differ between the COVID-19 and Post groups. Nor were significant differences found for perceived nightmares, χ^2^(4) = 7.49, *p* = 0.112, *Cramér's* V = 0.07. Nevertheless, Supplementary Table S1 shows that the Post group reported more anxious dreams and greater recall of dreams involving at least one scene.

Supplementary Table S1 also indicates significant intergroup differences in fear and/or anxiety caused by the dream, χ^2^(8) = 19.25, *p* = 0.014, Cramér's V = 0.11; recall quality, χ^2^(10) = 44.48, *p* < 0.001, Cramér's V = 0.12; and recurring dreams, χ^2^(8) = 28.36, *p* < 0.001, Cramér's V = 0.13. In contrast, the awakening stimulus did not differ significantly across groups, χ^2^(8) = 14.43, *p* = 0.071, Cramér's V = 0.07.

The absence of significant bivariate differences in nightmare frequency did not rule out the possibility that group differences could emerge after adjusting for relevant covariates. For this reason, several logistic regression models were tested with Nightmare as the dependent variable. Group (Pre, COVID-19, and Post), CEAD score, and dream recurrence were entered as independent variables, together with the relevant control variables.

All models showed low Nagelkerke *R*^2^ values. Group was not significant in any model, whereas the CEAD score was significant in all models. In several models, sleep medication was the only control variable significantly associated with nightmares. Because sleep medication was also significantly associated with nightmares in the bivariate analysis, Model 1 was retained to preserve continuity between the bivariate and multivariate findings (Supplementary Table S3). The model was statistically significant, χ^2^(7) = 63.2, *p* < 0.001, and showed good discrimination, AUC = 0.80, 95% CI [0.74, 0.85]. The Hosmer-Lemeshow test indicated adequate model fit, and the null hypothesis of good fit was therefore not rejected, χ^2^ = 2.50, df = 8, *p* = 0.962. Although the model was statistically significant, its explanatory power was modest (Nagelkerke *R*^2^ = 0.16), indicating that a substantial proportion of the variance in nightmares remained unexplained.

In Model 1 (Supplementary Table S3), higher AUA levels were associated with a higher likelihood of reporting nightmares. The maximum correlation between predictors was ±0.54, indicating that the high OR for the CEAD score, above 18, 95% CI [5.68, 60.70], was not attributable to multicollinearity but rather reflected a strong association between AUA and nightmares. Sleep medication was also significantly associated with nightmares, in line with the bivariate findings after the crisis. Recurring dream motifs were associated with 2.8- to 3.1-fold higher odds of reporting nightmares. Once the other predictors were controlled for, no significant group-related variation was observed in the likelihood of reporting nightmares.

Model 1 did not include COVID-19-related variables because these data were not available for the Pre group, which was assessed before the pandemic. Additional models were therefore explored using only the COVID-19 and Post groups, for which COVID-19-related variables were available. These models combined the COVID-19 data from the baseline study with the Post data from the present study. None of the COVID-19-related variables reached statistical significance, and no additional predictors improved Model 1. These results were consistent with the bivariate findings: membership in the COVID-19 group was not associated with higher nightmare frequency. Only the CEAD score, recurring dreams, and sleep medication remained associated with nightmares.

### 3.5 Linear regression model for anxiety upon awakening

A bootstrap-based analysis of variance indicated significant differences among the three groups, *F* = 10.85, *MS* = 1.55, *df* = 2, *p* < 0.001. According to the Scheffé test, these differences were attributable to higher CEAD scores in the Post group compared with the Pre and COVID-19 groups. These results did not support hypothesis H2 regarding a return to pre-pandemic anxiety values after the pandemic.

To examine whether this pattern remained after accounting for other variables, linear regression models were estimated. Model 2 (Supplementary Table S4) showed an *R* of 0.38 and a Durbin-Watson statistic of 2.04. Across the three groups, AUA was negatively associated with age, sleep medication, and sleep duration, and positively associated with having experienced a nightmare and with belonging to the COVID-19 or Post groups. Thus, AUA remained higher than in the Pre group at the time of this study. Although the model was statistically significant, its explanatory power was modest (*R*^2^ = 0.15), indicating that a substantial proportion of the variance in AUA remained unexplained.

Model 3 (Supplementary Table S4) added the COVID-19-related variables available for the COVID-19 and Post groups. The frequency and intensity of worry about the situation were positively associated with AUA, whereas frequent hand-washing showed a negative association. In addition, as observed in the bivariate analysis, the Post group showed higher AUA levels than the COVID-19 group. These findings again did not support hypothesis H2.

Model 3 also showed that insomnia was associated with higher AUA scores. Regarding dream characteristics, having had a nightmare or a recurring dream was also associated with higher AUA. The variables excluded from Model 3 are also informative. Belonging to a risk group, taking additional hygiene measures to avoid infection, and other sleep disorders, such as narcolepsy or restless legs syndrome, were not retained as significant predictors.

### 3.6 Path analysis

**Figure 1:**
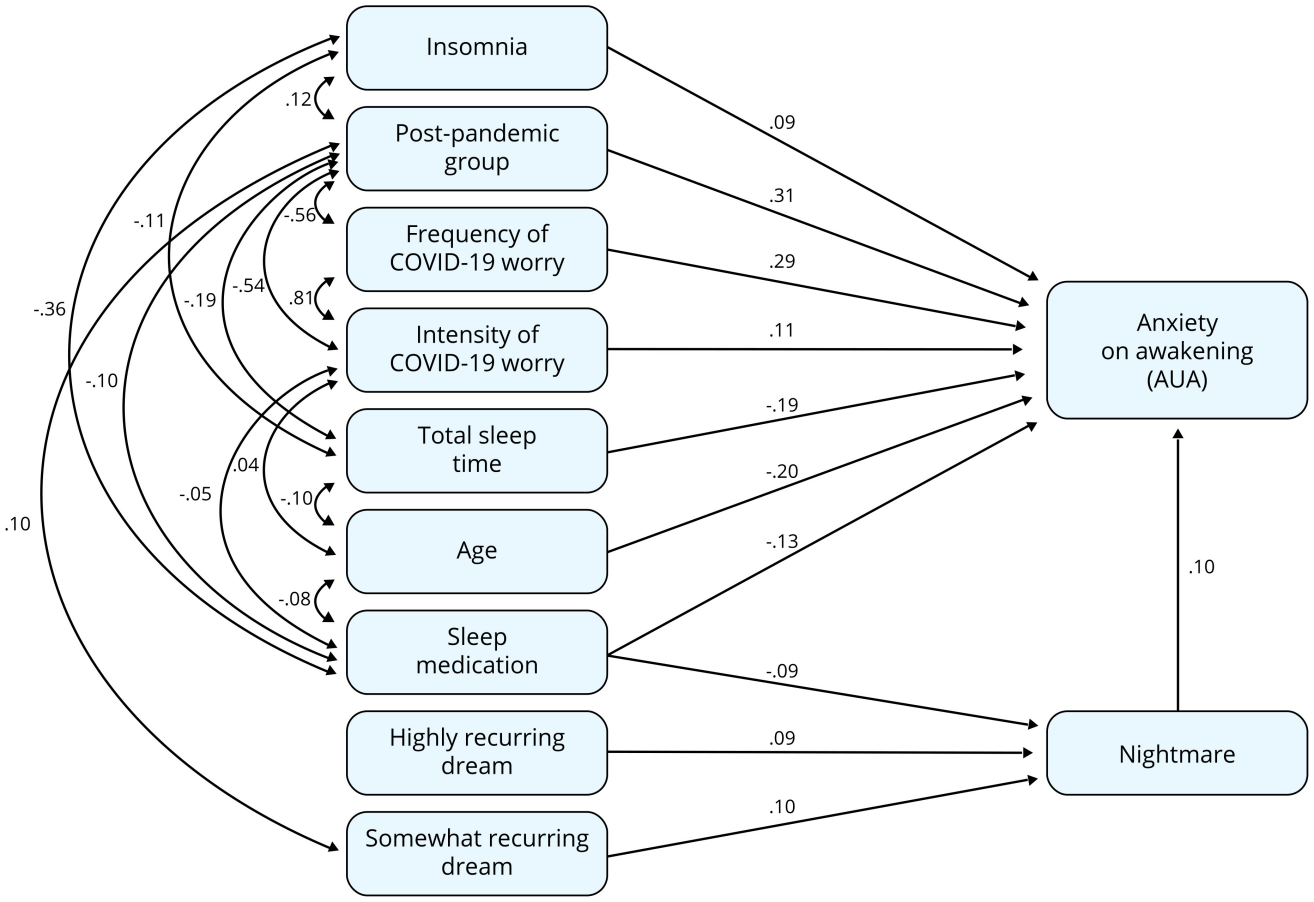
Path analysis integrating the significant variables from the regression models for the COVID-19 and post-pandemic groups. Only statistically significant paths are shown, and values on the arrows are standardized coefficients. AUA, anxiety upon awakening.

A path analysis was conducted to obtain an integrated view of the findings from the previous models. The analysis included the COVID-19 and Post groups and incorporated the relevant variables identified in the regression models. All independent variables were allowed to intercorrelate. A directional path from nightmares to AUA was specified on theoretical and temporal grounds, because the dream experience necessarily precedes the evaluation of anxiety at awakening. However, given the repeated cross-sectional design, this path should be interpreted as a theory-driven directional assumption rather than as direct evidence of causality. Using maximum likelihood estimation and progressively removing non-significant regression coefficients and correlations, the model shown in Figure 1 was obtained.

The model had a χ^2^ = 57.32 (*df* = 31, *p* = 0.003), *CMIN/DF* = 1.85, *CFI* = 0.99, *TLI* = 0.98 and *NFI* = 0.97. The *RMSEA* value was 0.03 (*LO 90* = 0.02, *HI 90* = 0.04). Although, in general, the goodness-of-fit indices are adequate, the χ^2^ test points to a possible poor fit to the data. It is not possible to rule out that this was due to the lack of multivariate normality of the data and the sample size.

In fact, Mardia's test of multivariate normality is high for the data (*c.r*. = 91.14), very likely due to the model containing some binary variables. The model was therefore recalculated using the distribution-free least squares discrepancy procedure. With this procedure, the goodness-of-fit indices were also acceptable (*GFI* = 0.99; *AGFI* = 0.98; *RMR* = 0.05; *NFI* = 0.97). As a result, these results indicate that the model provided an acceptable fit to the data.

In the model shown in Figure 1, sleep medication was negatively associated with both nightmares and AUA. Recurring dreams were associated with a higher likelihood of reporting a nightmare. AUA, in turn, was associated with younger age, shorter total sleep time, insomnia, belonging to the Post group rather than the COVID-19 group, and greater frequency and intensity of worry about COVID-19. The Post group was also characterized by lower use of sleep medication, shorter total sleep time, and lower COVID-19-related worry, but by more recurring dream elements.

## 4 Discussion

This study examined whether sleep, nightmares, and anxiety upon awakening (AUA) returned to pre-pandemic levels after the lifting of COVID-19 restrictions. The results point to a mixed pattern of recovery. Sleep duration returned to values similar to those observed before the pandemic, and worry about COVID-19 was lower than during lockdown. However, AUA remained elevated, particularly in the Post group, and dream activity showed greater emotional salience, with more anxious dream content, better dream recall, and more recurring elements. In contrast, operationally defined nightmare frequency remained low, with no clear variation across the Pre, COVID-19, and Post periods. Overall, these findings indicate that post-pandemic emotional activation may have been expressed more clearly through AUA and anxious dream experience than through an increase in nightmares as defined by the three criteria used in this study.

### 4.1 Sleep

With the return to normal life, sleep duration (Md = 6–7 h) did not differ from the Pre period, supporting hypothesis H1 of a return to pre-pandemic values. During lockdown, sleep duration had been significantly higher, with a median of between 7–8 h. This pattern indicates that sleep duration normalized after the lifting of restrictions.

Sleep-related variables were more clearly associated with AUA than with nightmares. Participants without sleep disorders presented lower AUA levels, whereas insomnia and restless legs syndrome were associated with higher AUA. This pattern points to an association between sleep quality and the emotional state reported at awakening. Sleep medication was also associated with a lower likelihood of reporting a nightmare, although this finding should be interpreted cautiously and not as evidence of a treatment effect.

However, neither sleep duration nor sleep disorders were associated with nightmares in the present study. This contrasts with previous studies in which poorer sleep quality, shorter sleep duration, or more awakenings were associated with nightmares or other negative dream-related experiences during the pandemic (Proença Becker et al., 2024; Simões et al., 2023; Solomonova et al., 2021).

### 4.2 Dreams and nightmares

It was not possible to reject hypothesis H3 that there would be no differences in nightmare frequency between the COVID-19 and Post groups. Several non-mutually exclusive reasons may help explain this pattern:

1. Nightmare frequency remained low in the Post group, at 4.6%, with no clear variation relative to the Pre and COVID-19 groups. Pairwise bootstrap estimates showed that the observed absolute differences between periods were small, all below 2 percentage points, although the corresponding confidence intervals indicated limited precision to rule out small differences completely. Perceived nightmares also did not differ significantly among the three periods.
2. In the Post group, more dreams caused anxiety, and dream recall was higher. However, this did not translate into higher nightmare frequency, because the operational definition required awakening caused by dream content, which remained stable across periods.
3. The threshold at which dream content awakens the sleeper may also have been modified by greater physiological sleep pressure, since a reduction in total sleep duration was observed in the sample. This interpretation should be regarded as tentative, given that sleep pressure was not directly measured in the present study. Even so, it fits with the hypothesis of a strong sleep homeostatic process.
4. Nor were associations observed in the multivariate models between nightmares and variables for which such relationships might have been expected, such as belonging to a COVID-19 risk group, the frequency of worry about the virus, engaging in behaviors to avoid infection, or sleep disorders such as insomnia or restless legs syndrome.
5. In addition, the more specific operational approach used here, together with the non-retrospective nature of data collection, likely reduced comparability with studies based on subjective classifications or retrospective data. It may also have reduced sensitivity to low-frequency events.

The absence of significant differences in nightmare frequency should not be interpreted as definitive evidence that no real differences existed in the study population. Because nightmares were infrequent, the statistical analysis had limited precision to exclude small differences between periods. Nevertheless, the observed between-group differences in the sample were small and did not show the type of clear increase that might be expected if nightmares were a highly sensitive population-level response to a large-scale stressor such as the COVID-19 pandemic. Therefore, the findings indicate no clear variation in nightmare frequency under the operational definition used here, while still allowing for possible modest differences that this study could not rule out with sufficient precision.

These results align with studies that did not find substantial changes in dream frequency, recall, content, emotionality, vividness, or perceived nightmares during lockdown (Koppehele-Gossel et al., 2023; Scarpelli et al., 2022; Schredl and Bulkeley, 2020). However, these findings should also be considered in light of studies reporting pandemic-related changes in dream content and emotionality, including COVID-19-related themes, perceived nightmares, illness and death content, contamination-related imagery, threat and chase themes, and negative emotions such as fear, sadness, anger, and frustration (Barrett, 2020; Mota et al., 2020; Proença Becker et al., 2024; Simões et al., 2023; Solomonova et al., 2021; Yang et al., 2023). Taken together, this mixed evidence indicates that pandemic-related dreaming may have varied depending on population characteristics and measurement approach, including whether studies assessed perceived nightmares, or dream emotionality.

This methodological variability is particularly relevant for nightmare prevalence. As noted in previous reviews, prevalence estimates vary substantially depending on whether studies use binary items, rating scales, or dream diaries, and on whether nightmares are defined only by emotional content or also require an awakening criterion (Schredl and Reinhard, 2011). The more specific operational approach used in the present study therefore differs from many previous studies and limits direct comparison with prevalence estimates based on broader or retrospective measures. Under this definition, nightmare frequency did not show a discernible shift following strict lockdown or after the lifting of restrictions. In this sample, nightmares may have been a less sensitive indicator of post-pandemic sleep-related emotional activation than AUA. This may be due to:

- Higher AUA could have been associated with a more disturbing evaluation of dreams, leading some otherwise ordinary dreams to be perceived as nightmares.
- Higher AUA could also have co-occurred with more threatening characteristics of dream characters (Saez-Uribarri, 2008), which may in turn have contributed to participants perceiving their dreams as nightmares. This may also help explain the increase in dreams that caused anxiety in this study.
- The emotional burden of pandemic-related dreaming may have been expressed not primarily through operationally defined nightmares, but through a broader range of dream manifestations, including anxious, defensive, compensatory, or metaphorical dream contents (Margherita et al., 2021; Monaco et al., 2022; Sacchetti et al., 2024).
- Methodological differences may also have contributed to divergence across studies. Perceived nightmares were twice as common as nightmares defined according to the three operational criteria.
- Greater insomnia and poorer sleep quality may have been associated with more awakenings, greater dream recall, and more perceived nightmares (Alfonsi et al., 2022; Casagrande et al., 2020; Simões et al., 2023). People with worse sleep quality also reported more negative affect in their dreams (Sikka et al., 2024; Westerberg et al., 2024).
- Cognitive biases during the crisis, such as expectations of more negative experiences during lockdown, may also have shaped the evaluation of some dreams as nightmares or directed greater attention to emotionally negative dreams.

### 4.3 Anxiety upon awakening

Both the COVID-19 and Post groups showed higher AUA levels than the Pre group, and the Post group also showed higher values than the COVID-19 group. Because the study used a repeated cross-sectional design with independent cohorts, this pattern should not be interpreted as longitudinal worsening in the same individuals. Rather, it indicates that emotional activation at awakening had not returned to pre-pandemic levels in this sample and may have remained particularly salient after the acute phase of the crisis.

The multivariate results indicated that higher scores on this construct were associated with a profile characterized by younger age, shorter sleep duration, insomnia, persistent worry about COVID-19, and dream experiences involving anxiety or nightmares. Sleep medication showed a negative association with AUA, but this finding should not be interpreted as evidence of a treatment effect. Rather, medication use may have acted as an indirect marker of sleep-related or clinical characteristics not fully captured in the present study.

Taken together, these findings point to a residual pattern of emotional vulnerability expressed at the sleep-wake transition, rather than a full return to pre-pandemic functioning. This interpretation remains tentative, given the model's modest explanatory power and the absence of several relevant contextual variables. More broadly, AUA may be understood both as an outcome associated with sleep- and dream-related emotional activation and as a potentially useful indicator for interpreting the subjective evaluation of dream experiences.

The results obtained are in line with other studies, which found fear, distress, or anxiety in dreams related to COVID-19 that occurred immediately before (MacKay and DeCicco, 2020) or during lockdown (Kilius et al., 2021; Proença Becker et al., 2024; Schredl and Bulkeley, 2020). Similarly, (Sacchetti et al. 2024) found that pandemic dreams were often dominated by fear and anxiety and, in aggressive interactions, more often placed the dreamer in the role of the victim, reflecting a sense of helplessness and threat associated with the pandemic context. Previous studies have also observed a relationship between content and AUA (Saez-Uribarri, 2008; Saez-Uribarri and Oberst, 2020).

### 4.4 Worry about COVID-19

As expected, worry about COVID-19 decreased with the return to normal life. In the bivariate analysis, worry was related to dreams involving fear or anxiety, operationally defined nightmares, perceived nightmares, and AUA. This pattern accords with previous studies linking worry, anxiety, and negative dream affect during the pandemic (Musse et al., 2020; Proença Becker et al., 2024; Simões et al., 2023; Sommantico and Parrello, 2023). It also parallels the findings of Sikka et al. (2024), who found that average daily worry, rather than day-to-day changes in worry, was associated with dream affect, suggesting that more worry-prone profiles may show greater vulnerability to disturbing dream experiences.

However, in the multivariate models of the present study, worry remained associated with AUA but not with nightmares. This suggests that COVID-19-related worry may have been reflected more clearly in the emotional state reported at awakening than in the occurrence of nightmares as defined by the three criteria used in this study. Therefore, worry may have contributed to a broader state of sleep-related emotional activation, without necessarily increasing the likelihood of awakening from a dream with fear or anxiety.

### 4.5 Sex and age

In terms of AUA with the return to normal life, a relationship was observed with sex, with higher AUA levels for women, as well as a negative relationship with age. In contrast, sex was not relevant in the multivariate models. These observations broadly align with previous findings on sex and age. With regard to sex, it was not found to be related to nightmares, dream content, or perceived changes in dreams (Abbas and Samson, 2023; Jiang et al., 2020; Loukola et al., 2024; Westerberg et al., 2024). Nevertheless, in other studies, it was observed that being a woman or being younger was associated with a higher frequency of reporting perceived nightmares, state anxiety, and pandemic content (Aknin et al., 2022; Musse et al., 2020; Simões et al., 2023; Solomonova et al., 2021).

### 4.6 Integration with theoretical frameworks

Nightmares did not show clear variation across the pandemic periods, either during lockdown or after the lifting of restrictions. One possible interpretation is that the emotional activation associated with dream content was usually not intense enough to interrupt sleep.

Sleep homeostasis has been described as an overwhelming physiological process regulating sleep pressure, duration, and intensity (Borbély and Tobler, 2024; Franks and Wisden, 2021). Within this framework, sleep tends to be preserved unless internal or external stimuli reach sufficient intensity to interrupt it. Homeostasis includes both restorative processes and anticipatory processes that prepare the organism for future demands, the latter being especially relevant in the second half of sleep, when REM sleep and more vivid emotional dreaming are more frequent (Borbély and Tobler, 2024; Simor et al., 2023). From this perspective, nightmares may be understood as relatively infrequent events in which emotionally intense dream content exceeds the threshold required to become an internal awakening stimulus. Fear and threat-related content are especially relevant in this regard, since fear is common in dreams and content involving death, aggression, or hostility has been linked to bad dreams and nightmares (Fireman et al., 2014; Nielsen et al., 1991). Fear and death-related content have also been reported in COVID-19 dreams (Barrett, 2020; Proença Becker et al., 2024; Yang et al., 2023). The absence of clear variation in nightmare frequency may therefore be interpreted as fitting the idea that, although emotional dream content increased, it did not more often reach the threshold required to overcome the sleep-preserving effect proposed by homeostatic theory.

In other words, the results on nightmares in this study do not provide clear support for theories that posit an adaptive function for nightmares in this context. Some reasons for this may be as follows:

- Disruption of adaptive cognitive and emotional processing.
- Incomplete processing of dream content, which may contribute to greater emotional distress.
- Worsening of sleep quality, with greater next-day fatigue.

Accordingly, the present results do not provide clear support for the proposed function of nightmares within Revonsuo's (2000) threat simulation theory, according to which the rehearsal of threat perception and threat avoidance in dreams represented an evolutionary advantage for human survival in ancestral environments. Within this framework, exposure to real threatening events, especially those involving risks to survival, might be expected to increase threatening dream content, particularly nightmares. As (Revonsuo 2000) argued, “nightmarish dreams are not ones that failed to perform their function, but, by contrast, are prime examples of the kind of dreams that fully realize their biological function,” and the risk of contracting a potentially serious illness could be understood as a threatening stimulus within this framework (Revonsuo and Valli, 2000). Nevertheless, the present results do not indicate that the threat simulation system was activated strongly enough to generate a population-level increase in nightmares. This interpretation should be balanced against studies that have reported support for the theory (Abbas and Samson, 2023; Yang et al., 2023). For example, (Loukola et al. 2024) interpreted the greater presence of illness-related threats in pandemic dreams as evidence of some activation of the threat simulation system, although pandemic dreams represented less than 12% of dreams in their study. According to these authors, the mechanism did not appear to be activated broadly enough to produce a clear effect on nightmare frequency.

In relation to the continuity hypothesis of dreaming (Domhoff, 1996; Schredl, 2003), the multivariate models in this study do not incorporate worry as a relevant variable for nightmares. As has been seen previously, the consideration of other variables such as AUA, sleep medication, and dream recurrence nullified its effect on nightmares. This observation agrees with the results obtained by Sikka et al. (2024), who also failed to find that daily changes in the worry of participants gave rise to changes in the affect of dreams; the relationship between nocturnal and diurnal events was due to individual differences. Mota et al. (2020) also observed greater vulnerability among subjects that was identifiable in reports. These findings provide limited support for the continuity hypothesis of dreaming in this specific context, although they do not rule it out and should be interpreted alongside studies showing that COVID-19-related dreams reflected waking concerns or emotions. Other researchers have reported findings more supportive of this framework (Barrett, 2020; MacKay and DeCicco, 2020; Pesonen et al., 2020; Sacchetti et al., 2024; Solomonova et al., 2021; Sommantico and Parrello, 2023; Yang et al., 2023).

According to emotional-processing accounts of dreaming (Nielsen et al., 1991; Nielsen and Lara-Carrasco, 2007), emotions in dreams may be as relevant as emotions experienced during wakefulness. Several studies have interpreted COVID-19-related dreams as contributing to the processing of fear and anxiety (Mota et al., 2020; Proença Becker et al., 2024; Solomonova et al., 2021). Similarly, Simões et al. (2023), although they did not directly compare their findings with pre-pandemic data, interpreted the frequency of negative emotions and affective states in COVID-19 dreams as supporting an emotional regulation account.

In the present study, the findings may be read within this theoretical framework, although they do not directly demonstrate emotional processing during sleep. First, the post-pandemic period was characterized by greater emotional load in dream experience, reflected in more dreams that caused anxiety and greater dream recall, without a parallel increase in nightmare frequency. In the Post sample, 39% of participants reported fear or anxiety caused by dream content, but only 4.6% met the operational definition of a nightmare. Second, this emotional load does not appear to have exceeded the awakening threshold more frequently. A nightmare required the simultaneous presence of three elements: recall of at least one dream scene, an emotional response of fear or anxiety, and awakening caused by dream content. Third, part of this emotional load may have been expressed at the sleep-wake transition, precisely where AUA was assessed. From this perspective, dream-related emotional activation may have been modulated during sleep and become consciously manifest upon awakening. However, this interpretation remains tentative, because emotional processing during sleep was not directly assessed in the present study.

### 4.7 Utility of the findings

Several authors have proposed that dream content may be useful for diagnostic assessment and/or psychological therapy in crises such as COVID-19. On the one hand, dream content may help identify potentially vulnerable individuals (Proença Becker et al., 2024; Schredl and Bulkeley, 2020), identify persistent mental health problems (Westerberg et al., 2024), and provide information about the patient's state of mind (Mota et al., 2020). On the other hand, dream-related variables have been discussed as potentially useful for promoting self-regulation in media exposure and developing strategies to increase coping efficacy (Guo and Shen, 2021).

Based on the results obtained, several tentative considerations can be added to proposals already advanced in the literature for future public health crises. These should not be understood as direct clinical recommendations, but as possible areas for future research and preventive monitoring:

- Strategies addressing nightmares, AUA, and insomnia could be explored as possible ways to identify or support profiles with greater emotional vulnerability during periods of collective stress.
- Sleep duration could be considered in crisis-monitoring protocols, since restricted sleep was associated with higher AUA in the present study.
- Recurring dream content may provide complementary information for monitoring persistent emotional activation, especially when accompanied by nightmares or elevated AUA.
- Worry-management strategies could be examined in crisis-response programs, given the association between COVID-19-related worry and AUA.
- More vulnerable groups could be considered in support or preventive-monitoring strategies during future public health crises; in the present sample, this possibility was supported by the higher AUA observed among younger participants.

### 4.8 Limitations of the study

The study has several limitations. First, the sample was not random and should not be considered representative of the general population. Women were overrepresented, participants were recruited through a Facebook advertisement, no socioeconomic indicators were collected, and the sample was restricted to Spain. These characteristics limit the generalizability of the findings beyond this sample and to other countries or cultural settings.

Second, the study used a repeated cross-sectional design with independent cohorts. Therefore, the results compare group-level patterns across sampling periods but cannot be interpreted as intraindividual change over time. The analyses identify associations, but they do not establish temporal directionality or causality.

Third, relevant contextual variables, such as social support, economic impact, physical health, work-related stress, personal relationships, and other stressful events unrelated to the pandemic, were not assessed. This may partly explain why the statistical models accounted for only part of the variability in the outcomes.

Fourth, the comparability of nightmare measures across studies is limited by the lack of consensus on how to define nightmares and distinguish them from anxious, negative, or disturbing dreams. Although the stricter three-component definition used here may reduce comparability with studies using broader or subjective classifications, it provides information on a more precisely defined phenomenon.

A fifth limitation concerns the self-reported nature of the sleep-related variables. Sleep duration was not assessed using objective measures such as actigraphy or polysomnography, and medication use and sleep disorders were not clinically verified within the study protocol. This should be considered when interpreting the sleep-related findings. In contrast, dream-related variables necessarily relied on participants' subjective recall, as is inherent to dream research. However, the present study reduced some of the limitations associated with unstructured dream reports by collecting the same information systematically across participants on the day of awakening and by operationalizing nightmares through three predefined criteria: recall of at least one dream scene, fear or anxiety caused by the dream, and awakening caused by dream content.

Finally, regarding recall bias, the study minimized this risk by collecting data on the same day as the dream and by recording the time elapsed between awakening and questionnaire completion. This interval was examined in the analyses and did not show significant associations with the main explanatory variables. Therefore, although any study based on dream recall cannot completely rule out memory-related effects, the present design reduces the limitations typically associated with retrospective dream reports.

## 5 Conclusion

The findings point to a mixed pattern of post-pandemic recovery. Sleep duration returned to pre-pandemic values, whereas anxiety upon awakening remained elevated and dream activity showed greater emotional salience, with more anxiety-related dreams and greater dream recall. This pattern indicates that the return to normal life was not accompanied by a full normalization of sleep-related emotional activation in this sample. In addition, no clear increase in nightmare frequency was observed during lockdown or after the lifting of restrictions within the stricter operational definition used here, which required dream recall, fear or anxiety, and awakening caused by dream content. Nightmares also did not emerge as a marker of post-pandemic sleep-related emotional activation as sensitive as anxiety upon awakening. Overall, the results support the value of examining anxiety upon awakening together with dream emotionality and nightmare frequency in future studies of sleep, dreams, and psychological adaptation after collective stressors.

## Data Availability

The raw data supporting the conclusions of this article will be made available by the author, without undue reservation.
