# Supplementary material for: The return to normal life: sleep, anxiety upon awakening, and nightmares following the crisis caused by the COVID-19 pandemic
Source: Front Sleep. 2026 Jun 24;5:1748727. doi: 10.3389/frsle.2026.1748727 (PMC13342196; doi:10.3389/frsle.2026.1748727)
Supplement: Supplementary file 1 [file Data_Sheet_1.pdf]

Questionnaire — Sleep and Dreams

EN-US section first, then Spanish (ES).

PART A — ENGLISH (US)

Information & Consent

The aim of this study is to learn about the effect of the Coronavirus crisis on sleep quality. The person responsible for this study is Iñigo Saez Uribarri, a licensed psychologist in Bizkaia, Spain (No. BI05070) and an independent researcher (Facebook: onirotest; email: inisaezu@yahoo.es). This research is not funded by any entity. The responsible party guarantees compliance with Spain's Law 14/2007 on Biomedical Research, Spain's Organic Law 3/2018 of December 5 on the Protection of Personal Data and Guarantee of Digital Rights, and Regulation (EU) 2016/679 of the European Parliament and of the Council of April 27, 2016 (General Data Protection Regulation). This questionnaire is voluntary, and your response implies your explicit consent to participate.

Instructions

Answer with the option that best describes how you woke up today. There are no right or wrong answers. Do not spend too long on any one question.

Section 1 of 6

Rate the following (scale 1–5).

Item	1	2	3	4	5
Calm	○	○	○	○	○
Serene	○	○	○	○	○
Tired	○	○	○	○	○
Disconcerted	○	○	○	○	○
Physical discomfort	○	○	○	○	○
Angry	○	○	○	○	○
Difficulty swallowing	○	○	○	○	○
Distressed	○	○	○	○	○
Nausea or dizziness	○	○	○	○	○
Sweaty hands or other parts of the body	○	○	○	○	○
Trembling (hands or legs)	○	○	○	○	○
Tense body	○	○	○	○	○
Rapid heartbeat	○	○	○	○	○
Feeling shortness of breath	○	○	○	○	○
Agitated breathing	○	○	○	○	○
Negative feelings	○	○	○	○	○
Trembling or shivering even though it is not very cold	○	○	○	○	○
Hot	○	○	○	○	○
Startled	○	○	○	○	○
Downcast/low	○	○	○	○	○
Crying	○	○	○	○	○
Upset	○	○	○	○	○
Nervous	○	○	○	○	○

Happy/content	○	○	○	○	○
Shouting	○	○	○	○	○

How long ago did you wake up?

☐ More than 12 hours ago
☐ More than 6 hours ago
☐ More than 3 hours ago
☐ More than 2 hours ago
☐ More than 1 hour ago
☐ More than half an hour ago
☐ Less than half an hour ago

Approximately how long were you sleeping before you woke up? (Do not count time spent in bed without sleeping.)

☐ More than 12 hours
☐ More than 11 hours
☐ More than 10 hours
☐ More than 9 hours
☐ More than 8 hours
☐ More than 7 hours
☐ More than 6 hours
☐ More than 5 hours
☐ More than 4 hours
☐ More than 3 hours
☐ More than 2 hours
☐ More than 1 hour
☐ 1 hour or less
☐ I don't know / I'm not sure

What woke you up?

☐ Alarm clock
☐ Bed partner
☐ Noise in my surroundings
☐ What I was dreaming about
☐ Something else

Tell us what you remembered of your dreams upon awakening

☐ I could not remember anything (go to Section 3)
☐ Only the impression of having been dreaming
☐ An image, sound, sensation, or smell (without a story)
☐ A simple scene / something was happening
☐ A dream with several scenes / several things were happening
☐ I remember several different dreams during the night

Section 2 of 6 (if you remember anything you dreamed)

Did what you were dreaming cause fear or anxiety?

- ☐ Fear
- ☐ Anxiety
- ☐ Fear and anxiety
- ☐ Another feeling
- ☐ I don't know

Was what you dreamed a nightmare?

- ☐ Yes
- ☐ No
- ☐ I don't know

Have you had this same dream on any other occasion?

- ☐ No
- ☐ Yes, some things are repeated
- ☐ Yes, it is almost identical to another dream I have had
- ☐ Yes, it is a dream that has repeated on several occasions
- ☐ I don't know

Please write what you remember of the dream:

____

____

Section 3 of 6 (for all participants)

Medication

Are you taking any medication?

- ☐ Yes (go to Section 4)
- ☐ No

Section 4 of 6 (if you are taking medication)

Medication details

Is the medication you are taking for sleep?

- ☐ Yes
- ☐ No

If you remember, please tell us which one:

____

Are you taking any other type of medication?

☐ Yes
☐ No

If you remember, please tell us which one:

____

Section 5 of 6 (for all participants)

Coronavirus crisis

How often have you felt worried about this crisis in the past day?

☐ Very frequently
☐ Frequently
☐ Sometimes
☐ Rarely
☐ Not at all

How intense is this worry for you?

☐ I feel very worried
☐ I feel quite worried
☐ I feel somewhat worried
☐ I feel a little worried
☐ I do not feel worried at all

Do you consider yourself to be in a risk group? (You may choose several answers.)

☐ Yes, due to age
☐ Yes, due to illnesses I have
☐ Yes, for other reasons
☐ I am not in a risk group

What precautions are you taking to avoid infection? (You may choose several answers.)

☐ I try not to leave my residence without an important reason
☐ I do not leave my residence by order of the authorities
☐ I use a mask, gloves, or alcohol gel
☐ I try not to touch surfaces that might be contaminated
☐ I wash my hands frequently
☐ I take other measures
☐ I do not take any precautionary measures

Has there been any death due to coronavirus in your environment?

☐ Yes, in my country
☐ Yes, in my region
☐ Yes, in my locality
☐ Yes, a friend or family member
☐ None has occurred

☐ I don’t know

Section 6 of 6 (for all participants)

Profile

Please indicate if your doctor has diagnosed you with any sleep disorder (you may choose several answers):

☐ Insomnia
☐ Sleep apnea
☐ Sleepwalking
☐ Restless legs syndrome
☐ Narcolepsy
☐ Other
☐ I have not been diagnosed with any

Are you male or female?

☐ Male
☐ Female

Date of birth (dd/mm/yyyy):

____

Place of birth (city or town):

____

Country of birth:

____

Country of residence:

____

Have you answered this form on another occasion?

☐ Yes
☐ No
☐ I don’t know

frontiers

PARTE B — ESPAÑOL

Información y consentimiento

El objetivo de este estudio es conocer el efecto de la crisis del Coronavirus en la calidad del sueño. La persona responsable de este estudio es Iñigo Saez Uribarri, psicólogo colegiado en Bizkaia, España (Nº BI05070) e investigador independiente (Facebook: onirotest; email: inisaezu@yahoo.es). Esta investigación no cuenta con financiación por parte de ninguna entidad. La parte responsable garantiza el cumplimiento de la Ley 14/2007 de Investigación Biomédica, la Ley Orgánica 3/2018, de 5 de diciembre, de Protección de Datos Personales y garantía de los derechos digitales, y del Reglamento (UE) 2016/679 del Parlamento Europeo y del Consejo, de 27 de abril de 2016 (RGPD). Este cuestionario es voluntario y su respuesta implica su consentimiento explícito para participar.

Instrucciones

Responda con la opción que mejor describa cómo se despertó hoy. No hay respuestas correctas o incorrectas. No dedique demasiado tiempo a cada pregunta.

Sección 1 de 6

Valore los siguientes ítems (escala 1–5).

Item	1	2	3	4	5
Tranquilo/a	○	○	○	○	○
Sereno/a	○	○	○	○	○
Cansado/a	○	○	○	○	○
Confuso/a	○	○	○	○	○
Malestar físico	○	○	○	○	○
Enfadado/a	○	○	○	○	○
Dificultad para tragar	○	○	○	○	○
Angustiado/a	○	○	○	○	○
Náusea o mareo	○	○	○	○	○
Sudoración (manos u otra zona)	○	○	○	○	○
Temblor (manos o piernas)	○	○	○	○	○
Tensión corporal	○	○	○	○	○
Latido acelerado	○	○	○	○	○
Falta de aire	○	○	○	○	○
Respiración rápida	○	○	○	○	○
Sentimientos negativos	○	○	○	○	○
Escalofríos (aunque no haga mucho frío)	○	○	○	○	○
Sensación de calor	○	○	○	○	○
Sobresaltado/a	○	○	○	○	○
Decaído/a	○	○	○	○	○
Llorando	○	○	○	○	○
Alterado/a	○	○	○	○	○
Nervioso/a	○	○	○	○	○
Feliz/Contento/a	○	○	○	○	○
Gritando	○	○	○	○	○

¿Hace cuánto se despertó?

☐ Más de 12 horas

☐ Más de 6 horas
☐ Más de 3 horas
☐ Más de 2 horas
☐ Más de 1 hora
☐ Más de media hora
☐ Menos de media hora

¿Cuánto durmió aproximadamente antes de despertar? (No cuente tiempo en cama sin dormir.)

☐ Más de 12 horas
☐ Más de 11 horas
☐ Más de 10 horas
☐ Más de 9 horas
☐ Más de 8 horas
☐ Más de 7 horas
☐ Más de 6 horas
☐ Más de 5 horas
☐ Más de 4 horas
☐ Más de 3 horas
☐ Más de 2 horas
☐ Más de 1 hora
☐ 1 hora o menos
☐ No lo sé / No estoy seguro/a

¿Qué le despertó?

☐ Despertador
☐ Persona con la que comparto dormitorio
☐ Ruido del entorno
☐ Lo que estaba soñando
☐ Otra causa

Cuéntenos qué recordó de sus sueños al despertar

☐ No recordé nada (pase a Sección 3)
☐ Solo la impresión de haber estado soñando
☐ Imagen, sonido, sensación u olor (sin historia)
☐ Una escena simple / algo sucedía
☐ Un sueño con varias escenas / varias cosas sucedían
☐ Recuerdo varios sueños distintos durante la noche

Sección 2 de 6 (si recuerda algo soñado)

¿Lo soñado le provocó miedo o ansiedad?

☐ Miedo
☐ Ansiedad
☐ Miedo y ansiedad
☐ Otro sentimiento

☐ No lo sé

¿Fue una pesadilla?

☐ Sí
☐ No
☐ No lo sé

¿Ha tenido este mismo sueño en otra ocasión?

☐ No
☐ Sí, se repiten algunas cosas
☐ Sí, casi idéntico a otro sueño
☐ Sí, es un sueño repetido en varias ocasiones
☐ No lo sé

Escriba lo que recuerde del sueño:

____

____

Sección 3 de 6 (para todas las personas)

Medicación

¿Está tomando alguna medicación?

☐ Sí (pase a Sección 4)
☐ No

Sección 4 de 6 (si toma medicación)

Detalles de la medicación

¿La medicación que toma es para dormir?

☐ Sí
☐ No

Si lo recuerda, indique cuál:

____

¿Toma otra medicación?

☐ Sí
☐ No

Si lo recuerda, indique cuál:

____

Sección 5 de 6 (para todas las personas)

Crisis del coronavirus

¿Cuántas veces ha sentido preocupación por esta crisis en el último día?

☐ Muy frecuentemente
☐ Frecuentemente
☐ A veces
☐ Rara vez
☐ En absoluto

¿Qué intensidad tiene esta preocupación para usted?

☐ Muy preocupado/a
☐ Bastante preocupado/a
☐ Algo preocupado/a
☐ Un poco preocupado/a
☐ Nada preocupado/a

¿Se considera grupo de riesgo? (puede marcar varias)

☐ Sí, por edad
☐ Sí, por enfermedades que padezco
☐ Sí, por otros motivos
☐ No soy grupo de riesgo

¿Qué precauciones toma para evitar el contagio? (puede marcar varias)

☐ Procuro no salir sin motivo importante
☐ No salgo por orden de las autoridades
☐ Uso mascarilla, guantes o gel
☐ Evito tocar superficies potencialmente contaminadas
☐ Me lavo las manos con frecuencia
☐ Tomo otras medidas
☐ No tomo medidas

¿Ha habido alguna muerte por coronavirus en su entorno?

☐ Sí, en mi país
☐ Sí, en mi región
☐ Sí, en mi localidad
☐ Sí, un amigo o familiar
☐ No ha ocurrido
☐ No lo sé

Sección 6 de 6 (para todas las personas)

Perfil

Indique si su médico le ha diagnosticado algún trastorno del sueño (marque todas las opciones que correspondan):

- ☐ Insomnio
- ☐ Apnea del sueño
- ☐ Sonambulismo
- ☐ Síndrome de piernas inquietas
- ☐ Narcolepsia
- ☐ Otro
- ☐ No tengo diagnóstico

¿Es hombre o mujer?

- ☐ Hombre
- ☐ Mujer

Fecha de nacimiento (dd/mm/aaaa):

____

Lugar de nacimiento (ciudad o pueblo):

____

País de nacimiento:

____

País de residencia:

____

¿Ha contestado este formulario en otra ocasión?

- ☐ Sí
- ☐ No
- ☐ No lo sé
