# Supplementary material for: The return to normal life: sleep, anxiety upon awakening, and nightmares following the crisis caused by the COVID-19 pandemic
Source: Front Sleep. 2026 Jun 24;5:1748727. doi: 10.3389/frsle.2026.1748727 (PMC13342196; doi:10.3389/frsle.2026.1748727)
Supplement: Supplementary file 2 [file Data_Sheet_2.pdf]

Supplementary material

Table S1. *Frequencies of Dream Characteristics by Sampling Time*

**Variables**	**Pre**	**COVID-19**	**Post**
Nightmare			
Yes	18	13	23
No	465	443	478
**Total**	**483**	**456**	**501**
Dream recall			
Could not remember anything	172 (1)	230 (2)	191
Only the impression of dreaming	70	60	55
An image, sound, sensation, or smell	39 (2)	22	22
A simple scene/something was happening	52	43	49
A dream with several scenes/several things were happening	101	59 (1)	107 (2)
I remember several different dreams during the night	49	42	77 (2)
**Total**	**483**	**456**	**501**
Awakening stimulus			
Alarm clock	129	104	132
The person who shares a bedroom with me	58	44	34
Noise in my surroundings	71	68	83
What I was dreaming of	39	33	41
Something else	176	207	211
**Total**	**473**	**456**	**501**
Fear and/or anxiety caused by the dream			
Fear	10	4	10
Anxiety	40 (1)	36	73 (2)
Fear and anxiety	38	32	38
Another feeling	149	97	139
I don’t know	73	57	50 (1)
**Total**	**310**	**222**	**310**
Perceived nightmare			
Yes	34	34	54
No	228	150	200
I don’t know	48	42	56
**Total**	**310**	**226**	**310**

Variables	Pre	COVID-19	Post
Recurring dream			
No	158	125	155
Yes, some things were repeated	81 (2)	28 (1)	72
Yes, almost identical to another dream I have had	14 (2)	5	5
Yes, a dream that I have had on several occasions	8	4	6
I don't know	49 (1)	64 (2)	72
**Total**	**310**	**226**	**310**

Note. (1) Adjusted standardized residual < –1.96 (frequency lower than expected, *p* < .05); (2) Adjusted standardized residual > +1.96 (frequency greater than expected, *p* < .05). Abbreviations: Pre = pre-pandemic period, COVID-19 = lockdown, Post = post-pandemic period. Data on fear and/or anxiety, perceived nightmares, and recurring dreams were only calculated for participants who recalled their dreams. Data for the Pre and COVID-19 groups came from the baseline study (Saez-Uribarri, 2020b).

Table S2. *ANCOVA on Total Sleep Duration*

Source	*SS*	*df*	*MS*	*F*	*p*	*Partial* η^2
Corrected model	71.62	4	17.90	20.27	< .001	.05
Intercept	20.47	1	20.47	23.18	< .001	.02
Age	25.03	1	25.03	28.33	< .001	.02
Sex	1.78	1	1.78	2.01	.156	.00
Group	55.36	2	27.68	31.34	< .001	.04
Error	1,253.36	1,419	0.88			
Total	1,324.98	1,424				
Corrected total	1,324.98	1,423				

Note. *SS* = Sum of squares; *df* = degrees of freedom; *MS* = mean square; partial η^2 = effect size. Data for the Pre and COVID-19 groups came from the baseline study (Saez-Uribarri, 2020b).

Table S3. *Factors Associated with the Occurrence of Nightmares According to Logistic Binary Regression*

							95% *CI for EXP(B)*	
Model 1	*B*	*SE*	*Wald*	*df*	*Sig.*	*Exp(B)*	Lower	Upper
Group			0.26	2	0.88			
Post Group	0.13	0.35	0.14	1	0.70	1.14	0.57	2.29
COVID-19 Group	0.09	0.40	0.06	1	0.81	1.10	0.50	2.41
Sleep medication	-1.00	0.42	5.68	1	0.02	0.37	0.16	0.84
Other medication	0.69	0.41	2.81	1	0.09	2.00	0.89	4.50
CEAD	2.92	0.60	23.38	1	< 0.01	18.57	5.68	60.70
Some recurring elements	1.03	0.34	9.21	1	< 0.01	2.79	1.44	5.41
Dream identical or almost identical to another	1.12	0.54	4.25	1	0.04	3.06	1.06	8.86
Constant	-6.09	1.10	30.49	1	< 0.01	0.00		

Note. *B* = unstandardized coefficient; *SE* = standard error; *Exp(B)* = odds ratio; *CI* = confidence interval; *df* = degrees of freedom; CEAD = Anxiety upon Awakening Assessment Questionnaire. Data for the Pre and COVID-19 groups came from the baseline study (Saez-Uribarri, 2020b).

Table S4. *Factors Associated with Anxiety upon Awakening According to Linear Regression*

Model 2	*Unstandardized Coefficients*		*Standardized Coefficients*	*t*	*Sig.*	*Multicollinearity Statistics*	
	B	*Std. Error*	*Beta*			*Tolerance*	*VIF*
(Constant)	1.28	0.10		13.43	< 0.01		
Age	-0.01	0.00	-0.25	-9.29	< 0.01	0.84	1.19
Sleep medication	-0.17	0.03	-0.14	-5.17	< 0.01	0.84	1.18
Taking another type of medication	-0.07	0.03	-0.08	-2.84	0.01	0.80	1.25
Sleep duration	-0.08	0.01	-0.21	-8.33	< 0.01	0.95	1.06
Nightmare	0.27	0.05	0.13	5.38	< 0.01	0.99	1.01
Post Group	0.14	0.02	0.17	5.65	< 0.01	0.68	1.48
COVID-19 Group	0.09	0.03	0.11	3.48	< 0.01	0.65	1.54
Model 3							
(Constant)	1.21	0.12		10.25	< 0.01		
Age	-0.01	0.00	-0.20	-6.87	< 0.01	0.93	1.07
Sleep medication	-0.14	0.04	-0.11	-3.40	< 0.01	0.75	1.34
Taking another type of medication	-0.03	0.03	-0.03	-0.97	0.33	0.79	1.26
Sleep duration	-0.08	0.01	-0.20	-6.77	< 0.01	0.91	1.10
Suffers insomnia	0.11	0.04	0.08	2.60	0.01	0.84	1.20
Nightmare	0.17	0.06	0.08	2.88	< 0.01	0.96	1.04
Recurring dream: some things repeated	0.09	0.04	0.07	2.43	0.02	0.96	1.04
Recurring dream: identical or almost identical to another dream	0.19	0.08	0.07	2.48	0.01	0.98	1.02
Post Group	0.20	0.03	0.26	6.99	< 0.01	0.56	1.80
Frequency of worry about COVID-19	0.13	0.02	0.30	6.00	< 0.01	0.32	3.14
Intensity of worry about COVID-19	0.05	0.02	0.11	2.20	0.03	0.32	3.12
I wash my hands frequently	-0.05	0.02	-0.06	-2.03	0.04	0.89	1.13

Note. *B* = unstandardized coefficient; *Std. Error* = standard error for the unstandardized coefficient; *Beta* = standardized coefficient; *t* = value of the *t* statistic; Sig. = significance level (*p*); Tolerance = tolerance (multicollinearity); *VIF* = variance inflation factor (multicollinearity). Data for the Pre and COVID-19 groups came from the baseline study (Saez-Uribarri, 2020b).

Table S5. *Weighted CEAD item scores assigned to each response category*

Item (English)	Original item in Spanish	Response category selected				
		1	2	3	4	5
Calm	Calmado/a	0.81	0.44	0.09	-0.25	-0.64
Disconcerted	Desconcertado/a	-0.39	0.13	0.39	0.73	1.17
Physical discomfort	Molestias físicas	-0.36	-0.07	0.12	0.31	0.56
Rapid heartbeat	Latidos rápidos del corazón	-0.33	0.32	0.63	0.94	1.31
Angry	Enfadado/a	-0.30	0.34	0.60	0.87	1.26
Distressed	Acongojado/a	-0.31	0.30	0.57	0.88	1.27
Sweaty hands or other parts of the body	Sudor en manos o en otra parte del cuerpo	-0.15	0.42	0.55	0.74	1.04
Tense body	El cuerpo en tensión	-0.44	0.13	0.40	0.69	1.11
Upset	Alterado/a	-0.35	0.52	0.84	1.19	1.73
Feeling shortness of breath	Sensación de falta de aire	-0.22	0.48	0.73	0.96	1.29
Negative feelings	Sentimientos negativos	-0.45	0.12	0.40	0.68	1.06
Hot	Acalorado/a	-0.27	0.16	0.36	0.59	0.87
Serene	Sereno/a	0.80	0.41	0.07	-0.25	-0.62
Startled	Sobresaltado/a	-0.33	0.43	0.73	1.03	1.45
Crying	Llorando	-0.12	0.68	0.87	1.08	1.41
Agitated breathing	Respiración agitada	-0.21	0.55	0.81	1.02	1.35
Nervous	Nervioso/a	-0.46	0.26	0.63	1.06	1.59
Shouting	Gritando	-0.08	0.62	0.77	0.89	1.06
Trembling or shivering even though it is not very cold	Tiritar o con escalofríos aunque no haga mucho frío	-0.17	0.35	0.69	0.95	1.26

Note. For each item, the CEAD score corresponds to the value assigned to the selected response category. The total CEAD score is calculated by summing these values across the 19 items. Higher total scores indicate greater anxiety upon awakening. Table S5 shows only the 19 items used to compute the abridged CEAD score analyzed in the manuscript.
